# Dual-channel hypergraph convolutional network for predicting herb–disease associations

**DOI:** 10.1093/bib/bbae067

**Published:** 2024-02-29

**Authors:** Lun Hu, Menglong Zhang, Pengwei Hu, Jun Zhang, Chao Niu, Xueying Lu, Xiangrui Jiang, Yupeng Ma

**Affiliations:** The Xinjiang Technical Institute of Physics and Chemistry, Chinese Academy of Sciences, Urumqi China; University of Chinese Academy of Sciences, Beijing, China; Xinjiang Laboratory of Minority Speech and Language Information Processing, Urumqi, China; The Xinjiang Technical Institute of Physics and Chemistry, Chinese Academy of Sciences, Urumqi China; University of Chinese Academy of Sciences, Beijing, China; Xinjiang Laboratory of Minority Speech and Language Information Processing, Urumqi, China; The Xinjiang Technical Institute of Physics and Chemistry, Chinese Academy of Sciences, Urumqi China; University of Chinese Academy of Sciences, Beijing, China; Xinjiang Laboratory of Minority Speech and Language Information Processing, Urumqi, China; The Xinjiang Technical Institute of Physics and Chemistry, Chinese Academy of Sciences, Urumqi China; University of Chinese Academy of Sciences, Beijing, China; Xinjiang Laboratory of Minority Speech and Language Information Processing, Urumqi, China; University of Chinese Academy of Sciences, Beijing, China; State Key Laboratory Basis of Xinjiang Indigenous Medicinal Plants Resource Utilization, Key Laboratory of Chemistry of Plant Resources in Arid Regions, Xinjiang Technical Institute of Physicsand Chemistry,Chinese Academy of Sciences Urumqi, China; University of Chinese Academy of Sciences, Beijing, China; State Key Laboratory Basis of Xinjiang Indigenous Medicinal Plants Resource Utilization, Key Laboratory of Chemistry of Plant Resources in Arid Regions, Xinjiang Technical Institute of Physicsand Chemistry,Chinese Academy of Sciences Urumqi, China; State Key Laboratory of Drug Research, Shanghai Institute of Materia Medica,Chinese Academy of Sciences Shanghai, China; The Xinjiang Technical Institute of Physics and Chemistry, Chinese Academy of Sciences, Urumqi China; University of Chinese Academy of Sciences, Beijing, China; Xinjiang Laboratory of Minority Speech and Language Information Processing, Urumqi, China

**Keywords:** herb–disease association prediction, network pharmacology, hypergraph convolutional network, multi-target multi-component, Chinese traditional medicine

## Abstract

Herbs applicability in disease treatment has been verified through experiences over thousands of years. The understanding of herb–disease associations (HDAs) is yet far from complete due to the complicated mechanism inherent in multi-target and multi-component (MTMC) botanical therapeutics. Most of the existing prediction models fail to incorporate the MTMC mechanism. To overcome this problem, we propose a novel dual-channel hypergraph convolutional network, namely HGHDA, for HDA prediction. Technically, HGHDA first adopts an autoencoder to project components and target protein onto a low-dimensional latent space so as to obtain their embeddings by preserving similarity characteristics in their original feature spaces. To model the high-order relations between herbs and their components, we design a channel in HGHDA to encode a hypergraph that describes the high-order patterns of herb-component relations via hypergraph convolution. The other channel in HGHDA is also established in the same way to model the high-order relations between diseases and target proteins. The embeddings of drugs and diseases are then aggregated through our dual-channel network to obtain the prediction results with a scoring function. To evaluate the performance of HGHDA, a series of extensive experiments have been conducted on two benchmark datasets, and the results demonstrate the superiority of HGHDA over the state-of-the-art algorithms proposed for HDA prediction. Besides, our case study on Chuan Xiong and Astragalus membranaceus is a strong indicator to verify the effectiveness of HGHDA, as seven and eight out of the top 10 diseases predicted by HGHDA for Chuan-Xiong and Astragalus-membranaceus, respectively, have been reported in literature.

## INTRODUCTION

As traditional Chinese medicines, herbs have been in existence for thousands of years, and they are widely used to treat and prevent complex diseases with accumulated valuable medication experiences [1]. In contrast to modern drugs that are composed of single components and designed to act on single target protein, herbs contain a variety of plant components, thus making them active on multiple molecular biological mechanisms simultaneously in cells. It is for this reason that herbs are effective in different diseases from a systematic perspective [2]. However, the main difficulty lying in the understanding of herb–disease associations (HDAs) is the multi-target and multi-component (MTMC) mechanism, which indicates the inherent complexity of herbs for botanical therapeutics. In this regard, there is a necessity for us to take into account the MTMC mechanism for improved performance of HDA prediction, and this challenging task is yet to be solved. Recently, the systemic concept of network pharmacology has been attracting much attention to facilitate the exploration of herbal mechanisms on the treatment of different diseases, and it provides a novel perspective for HDA prediction by constructing heterogeneous information networks with different related associations, including but not limited to herb-component, component–target protein and target protein–disease associations [3–5].

By leveraging network pharmacology in conjunction with existing traditional herb databases, the task of HDA prediction holds significant practical implications for clinical medication and formulation development. Numerous studies have established associations between certain herbs, including Astragalus, Chuanxiong, Ginseng and Salvia miltiorrhiza, and a range of potential diseases. For instance, Astragalus membranaceus has demonstrated efficacy in treating immune system and liver diseases [6–8], Ligusticum chuanxiong has shown promise in addressing neurological disorders [9], Panax ginseng has been linked to cardiovascular disease management [10] and Salvia miltiorrhiza has been indicated for cerebrovascular diseases [11]. Moreover, novel Chinese herbal medicines, such as Si-Ni-San, have exhibited therapeutic potential across multiple diseases [12–14].

Currently, several computational algorithms have been developed for association prediction in various domains, including drug–target protein association prediction [15–17], drug–disease association prediction [18–20] and protein–protein interaction prediction [21–24]. In the context of biological association prediction methods related to network pharmacology, three main categories are commonly employed, and they are similarity-based methods, reverse docking methods and network-based methods [25].

Similarity-based methods rely on measuring the sequence or structural similarity between molecules to predict associations, and they assume that molecules with similar structures may have similar biological functions [26–30]. For instance, drugCIPHER [29] combines pharmacology and genomic information, and it calculates therapeutic and chemical structure similarities between drugs to predict potential target proteins. Although similarity-based methods can quickly retrieve information about molecules, their performance considerably degrades when the number of known target proteins is limited, since they only consider information about individual molecules without utilizing relationships between them. Considering both molecular components and target protein information, reverse molecular docking targets to identify potential target proteins for a given small molecule or ligand through an opposite process of traditional molecular docking. However, they are not suitable for target proteins with unclear 3D structures, and require substantial computational resources [31–34]. An example of a reverse docking method is TarFisDock [31], which includes a program that performs reverse ligand–protein docking to identify potential binding proteins for a given small molecule.

Network-based methods leverage molecular association networks to learn embeddings of herbs and target protein molecules, thus enabling association prediction [35–40]. For instance, HTInet [35] constructs a heterogeneous information network that incorporates herbs, diseases, symptoms and target proteins. Node2vec, a graph embedding technique, is then utilized to learn low-dimensional representations of herbs and target proteins in the network, which are subsequently employed to predict potential herb–target protein associations. Another example is HGNA-HTI [36], which constructs a herb–target protein heterogenous network and employs a heterogenous graph neural network with attention and message-passing mechanisms to learn embeddings of herbs and target proteins for association prediction. Network-based methods provide the advantage of utilizing information from individual molecules and their associations simultaneously, and are relatively straightforward to implement. However, for new herbs lacking herb–target protein associations, additional information, such as similarity analysis, may still be required.

Most existing methods primarily focus on predicting component–target protein or herb–target protein associations, with limited attention given to associations between herbs and diseases. Additionally, these methods often fail to effectively utilize the MTMC properties of herbs. To address these limitations and better understand the multi-to-multi relationships between herbs and diseases, we propose a novel computational model called HGHDA (Hypergraph-based herb–disease association prediction). HGHDA introduces a dual-channel hypergraph convolution network to properly reveal the complicated mechanism inherent in the MTMC botanical therapuetics, thus achieving the task of HDA prediction from a high-level perspective of network pharmacology.

In particular, HGHDA begins by calculating similarity scores between components using their Canonical SMILES (Canonical Simplified Molecular Input Line Entry System) information, which provides a standardized representation of the chemical structures of herbal components. By comparing the Canonical SMILES of different components, HGHDA generates a similarity matrix that quantifies the similarity between each pair of components. Similarly, it also generates another similarity matrix of target proteins by calculating their similarity scores based on protein sequence information. An autoencoder is then employed by HGHDA to produce the embeddings of components and target proteins using their respective similarity matrices. The embeddings generated by the autoencoder aim to capture the essential features from the original feature spaces of components and target proteins.

**Figure 1:**
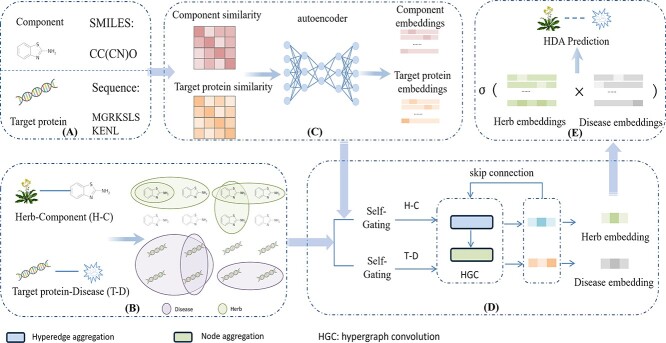
Overview of the model. (a) is data collection, (b) is the construction of hypergraphs, (c) is the acquisition of Compact Embeddings of Components and Target proteins, (d) is the acquisition of embeddings of herb and diseases, and (e) is the prediction of potential HDAs.

To obtain the embeddings of herbs and diseases, HGHDA incorporates two channels. The first channel represents the relationship between herbs and their components as a hypergraph, and employs the hypergraph convolution to capture high-order patterns that depict the multi-component property of herbs. The second channel represents the high-order relation patterns between diseases and their target proteins in a similar hypergraph manner. By aggregating the embeddings of herbs and diseases learned through dual-channels, HGHDA is able to encode the association information in consistence with the MTMC properties. Finally, HGHDA utilizes the learned embeddings of herb and disease as input of a scoring function, and predict potential associations between herbs and diseases. To evaluate the performance of HGHDA, we have conducted a series of extensive experiments on two benchmark datasets. Results demonstrate the superior performance of HGHDA over several state-of-the-art prediction models. Our case study on Chuan Xiong and Astragalus membranaceus also indicates that HGHDA is a promising tool to identify novel diseases for herbs. The overall framework of HGHDA is depicted in Figure 1.

## MATERIALS AND METHOD

### Datasets

In order to construct relevant hypergraphs and evaluate the performance of the HGHDA model, we adopt two benchmark datasets, one is collected from the TCM-suite database [41], and the other is curated from taxonomically related plants [42]. Specifically, the first benchmark dataset, namely TCM-suite, is composed of herb–component associations, component–target protein associations and target protein–disease associations collected from two sub-databases: Holmes-Suite focusing on herb ingredient identification, and Watson-Suite facilitating pharmacological network analysis. From Table 1, a total of 1009 herbs, 1193 components, 7258 target proteins, 11,071 diseases, 6496 herb-component associations and 44 170 target protein–disease associations are collected, and they represent the relationships among herbs, components, target proteins and diseases in the database. There is a potential for 11 170 639 associations between 1009 herbs and 11 071 diseases. After integrating data on herb–compound associations, compound–target protein associations and target protein–disease associations, we have identified a total of 2354 225 known connections between all herbs and diseases, or HDAs.

**Table 1:** Data details in Dataset TCM-suite and ethnobotany

Dataset	Heterogeneous network properties	Amount
TCM-suite	Herb	1009
	Component	1193
	Target protein	7258
	Disease	11 071
	Herb-Component	6496
	Target protein-Disease	44 170
	Herb-Disease	2354 225
Ethnobotany	Herb	3039
	Component	40 933
	Target protein	355
	Disease	909
	Herb-Component	122 342
	Target protein-Disease	92 718
	Herb-Disease	16 234

The second benchmark dataset, Ethnobotany, is derived from a comprehensive cross-cultural analysis of traditional medicine, focusing on assessing the empirical and non-random nature of the traditional use of plants for medicinal purposes [42]. However, given that the original dataset lacks associations between diseases and target proteins, we integrate these associations from the Comparative Toxicogenomics Database [43], enabling the construction of a comprehensive hypergraph for the Ethnobotany dataset. Further statistical details about the Ethnobotany dataset are provided in Table 1.

All known HDAs are regarded as positive samples in the benchmark datasets. However, considering that the number of HDAs is significantly lower than the total number of herb–disease pairs in the benchmark datasets, we randomly choose herb–disease pairs without known associations to form a negative dataset of equal size to the positive samples. This selection enables us to create a negative dataset that matches the size of the positive samples, effectively mitigating any issues related to dataset imbalance.

### Constructing similarity matrices of components and target proteins

In order to calculate the similarity between herbal components, we use their Canonical SMILES information collected from the PubChem database [44]. For similarity calculations, the RDKit tool [45] is employed. Specifically, when converting the SMILES data of herb components into fingerprints, we use a configuration with 2048 bits for the fingerprints, which composed of elements representing specific substructures present in the molecule. When quantifying the similarity between two Morgan fingerprints, we utilize the Dice coefficient, which measures the overlap between the substructures represented by the fingerprints. Assuming that $c_{i}$ and $c_{j}$ are two herbal components, the Dice coefficient between them is computed as 


(1) \begin{align*}& \mathrm{\mathbf{S}^{ij}_{C}} =\frac{\mid M_{i} \cap M_{j} \mid}{(\mid M_{i} \mid+\mid M_{j} \mid)/2},\end{align*}

where $M_{i}$ and $M_{j}$ are the Morgan fingerprints of $c_{i}$ and $c_{j}$, respectively. A similarity matrix $\mathbf{S}_{C} \in \mathbb{R}^{n_{c} \times n_{c}}$, where $n_{c}$ is the number of components, can thus be obtained for indicating the similarity between pairwise components.

Regarding the similarity matrix between target proteins, we first collect the sequence information of target proteins from the Ensembl database [46], resulting in a set of target protein sequences. Subsequently, the PairwiseAligner module from the Biopython tool [47] is employed to calculate the similarity between target protein sequences. The parameters used in learning target protein similarity are set with their default setting provided in Biopython tool. Assuming that $n_{t}$ is the number of target proteins, we can finally construct a similarity matrix, denoted as $\mathbf{S}_{T} \in \mathbb{R}^{n_{t} \times n_{t}}$, thus enabling further analysis and modeling within the HGHDA framework.

### Learning compact embeddings of components and target proteins

Due to the issue of high dimensionality in $\mathbf{S}_{C}$ and $\mathbf{S}_{T}$, it is improper for us to explicitly use them as the feature vectors of components and target proteins. In particular, we employ the Autoencoder [48] model to obtain the compact embeddings of components and target proteins, and the parameters used to train Autoencoder are learning rate and epoch. Specifically, an autoencoder is utilized as an unsupervised learning model based on deep neural network, and it consists of an encoder and a decoder. The autoencoder is designed to compress the row vectors of $\mathbf{S}_{C}$ and $\mathbf{S}_{T}$ into lower dimensional embeddings. For instance, let $\mathbf{S}_{C}^{i}$ be the $i$-th row vector of $\mathbf{S}_{C}$, the encoding process can be represented as 


(2) \begin{align*}& \mathbf{W}_{C} =g(\mathbf{S}_{C}^{i}\mathbf{W}_{enc}+\boldsymbol{b}_{enc}),\end{align*}

where $\mathbf{W}_{enc}$ is the weight matrix, $\boldsymbol{b}_{enc}$ is the bias vector of the encoder and $g(\cdot )$ is a nonlinear activation function, such as RELU or Tanh. With (2), we are able to map the high-dimensional input vector $\mathbf{S}_{C}^{i}$ to a lower dimensional latent representation $\mathbf{W}_{C}$.

The decoder in the autoencoder is responsible for reconstructing $\mathbf{S}_{C}$ from $\mathbf{W}_{C}$. To do so, it performs the inverse transformation of the encoder, and attempts to reconstruct $\mathbf{S}_{C}$ as accurately as possible. The decoding process can be expressed as 


(3) \begin{align*}& \tilde{\mathbf{S}}_{C}^{i} =g(\mathbf{W}_{C}\mathbf{W}_{dec}+\boldsymbol{b}_{dec}),\end{align*}

where $\mathbf{W}_{dec}$ is the weight matrix, $\boldsymbol{b}_{dec}$ is the bias vector and $\tilde{\mathbf{S}}_{C}^{i}$ is the reconstructed version of $\mathbf{S}_{C}^{i}$. To approximate $\mathbf{S}_{C}^{i}$, we intend to minimize the error between $\tilde{\mathbf{S}}_{C}^{i}$ and $\mathbf{S}_{C}^{i}$ using the Mean Squared Error (MSE) as the loss function. The definition of MSE is presented as below. 


(4) \begin{align*}& L(\mathbf{S}_{C}^{i},\tilde{\mathbf{S}}_{C}^{i}) =\|\mathbf{S}_{C}^{i}-\tilde{\mathbf{S}}_{C}^{i}\|^{2}\end{align*}

In the training process of the autoencoder, a back-propagation algorithm is used to update the trainable parameters for minimizing the reconstruction error, which is defined as the average loss between the original component similarity vectors $\mathbf{S}_{C}^{i}$ and their reconstructed counterparts $\tilde{\mathbf{S}}_{C}^{i}$ according to (5). 


(5) \begin{align*}& \min\frac{1}{n}\sum_{i=1}^{n}L(\mathbf{S}_{C}^{i},\tilde{\mathbf{S}}_{C}^{i})\end{align*}

In the above equation, $n$ is the number of components involved in the dataset. The autoencoder is trained separately using $\mathbf{S}_{C}$ and $\mathbf{S}_{T}$ as inputs. The training process aims to learn $\mathbf{W}_{C}$ and $\mathbf{W}_{T}$, which are the embedding matrices of components and target proteins, respectively. By retaining the information consistent with the original feature space, the embeddings in $\mathbf{W}_{C}$ and $\mathbf{W}_{T}$ capture important characteristics and patterns of components and target proteins, serving as valuable input features utilized in the hypergraph convolution of the HGHDA model.

### Dual-channel hypergraph convolution

Given herb–component and target protein–disease associations, we formulate two association matrices denoted as $\mathbf{A}_{ch}$ and $\mathbf{A}_{td}$, which are then used as input to construct hypergraphs. According to [49], a hypergraph is a mathematical structure that generalizes the concept of a graph. In a standard graph, we have nodes and edges that connect pairs of nodes. In contrast, a hypergraph allows for edges to connect more than two nodes, which makes it a more flexible and expressive data structure. In our work, the hypergraph is primarily composed of components and target proteins. Herbs and diseases are treated as the edges of this hypergraph. Consequently, a herb hyperedge connects the components associated with that herb, while a disease hyperedge connects the target protein associated with that disease. A hypergraph is represented as $G = (V, \varepsilon , \mathbf{A})$, where

-
$V=\{v_{i}\}$ represents the set of nodes, with a length of $m$;
-
$\varepsilon =\{e_{j}\}$ represents the set of hyperedges, with a length of $n$;
-
$\mathbf{A} \in \{0,1\}^{m \times n}$ is an indication matrix used to represent the presence or absence of nodes and hyperedges in the hypergraph. Each element $a(v_{i}, e_{j})$ in $\mathbf{A}$ indicates whether a node $v_{i}$ exists in a hyperedge $e_{j}$.

In other words, the indication matrix $\mathbf{A}$ encodes the relationships between nodes and hyperedges in $G$. If $a(v_{i}, e_{j}) = 1$, it signifies that $v_{i}$ is part of $e_{j}$, while $a(v_{i}, e_{j}) = 0$ indicates that $v_{i}$ is not associated with $e_{j}$. Hence, according to the definition of $G$, we are able to construct two hypergraphs, i.e. $G_{ch}=\{V_{C},\varepsilon _{C},\mathbf{A}_{ch}\}$ and $G_{td}=\{V_{T},\varepsilon _{T},\mathbf{A}_{td}\}$. By representing herb–component and target protein–disease associations in the form of a hypergraph, HGHDA can capture and utilize the complex relationships involving multiple nodes. This enables HGHDA to effectively consider the MTMC properties between herbs and components, as well as target proteins and diseases, contributing to more accurate HDA prediction.

To jointly learn the embeddings of herbs and diseases, the HGHDA model employs a dual-channel hypergraph convolutional network. Each channel in the network performs a hypergraph convolution operation, which consists of two steps: hyperedge aggregation and node aggregation. Taking the herb channel as an example, the details of hypergraph convolution on $G_{ch}$ is present as below.

#### Hyperedge aggregation

In this step, HGHDA aggregates the information from $V_{C}$ to update the embeddings of $\varepsilon _{C}$. For each hyperedge, the model gathers information from component nodes within it, and then combines such information to generate a hyperedge embedding, which captures the higher order patterns and interactions among component nodes in the same hyperedge. To perform node-hyperedge transformation, the hyperedge aggregation is defined as 


(6) \begin{align*}& \mathbf{M}_{ch}=\mathbf{D}_{ch_{e}}^{-1}\mathbf{A}_{ch}^{T}\mathbf{E}_{ch},\end{align*}

where $\mathbf{D}_{ch_{e}}$ is the edge degree matrix of $\mathbf{A}_{ch}$, and $\mathbf{E}_{ch}$ and $\mathbf{M}_{ch}$ are, respectively, the component and hyperedge embedding matrices at the $l$-th layer. According to (6), the message passing from component nodes to their hyperedges is achieved by the multiplication operation $\mathbf{A}_{ch}^{T}\mathbf{E}_{ch}$. The purpose of using $\mathbf{D}_{ch_{e}}$ is to simply re-scale hyperedge embeddings.

#### Node aggregation

After obtaining $\mathbf{M}_{ch}$ with (6), HGHDA performs node aggregation to update the embeddings of component nodes by combining the embedding information of $\mathbf{E}_{ch}$ and $\mathbf{M}_{ch}$. This hyperedge-node transformation process allows HGHDA to capture the global context in $G$, and its definition is given as 


(7) \begin{align*}& \mathbf{N}_{ch}=\mathbf{D}_{ch_{v}}^{-1}\mathbf{A}_{ch}\mathbf{M}_{ch},\end{align*}

where $\mathbf{D}_{ch_{v}}$ is the node degree matrix of $\mathbf{A}_{ch}$ to re-scale component embeddings. With (7), premultiplying $\mathbf{A}_{ch}$ with $\mathbf{M}_{ch}$ is achieved to aggregate information from hyperedges to component nodes.

#### Hypergraph convolution

Combining (6) and (7), a complete hypergraph convolution is defined by (8). 


(8) \begin{align*}& \mathbf{E}^{l+1}=\mathbf{D}_{v}^{-1}\mathbf{A}\mathbf{D}_{e}^{-1}\mathbf{A}^{T}\mathbf{E}^{l}\end{align*}

However, it is possible for HGHDA to encounter the problem of feature information dilution when fusing multi-level aggregation in the learning process. In this regard, a resnet-lik skip connection [50] is integrated into HGHDA to avoid information dilution with many additional connections. Then, a transformed hypergraph convolution is defined as 


(9) \begin{align*}& \mathbf{E}^{l+1}=\sigma(\mathbf{D}_{ch_{v}}^{-1}\mathbf{A}_{ch}\mathbf{D}_{ch_{e}}^{-1}\mathbf{A}_{ch}^{T}\mathbf{E}_{ch}^{l}\Theta^{l}+\mathbf{E}_{ch}^{l}),\end{align*}

where $\Theta ^{l}$ is a trainable parameter and $\sigma (\cdot )$ is a nonlinear activation function, such as $ReLU(\cdot )$, $\mathbf{E}_{ch}^{0}=\mathbf{W}_{C}$ and $\mathbf{E}_{td}^{0}=\mathbf{W}_{T}$. With (9), HGHDA is able to simultaneously take into account original features and aggregated embeddings for generating the final representation of herbs.

By replacing $\mathbf{A}_{ch}$, $\mathbf{D}_{ch_{v}}$, $\mathbf{D}_{ch_{e}}$ and $\mathbf{E}_{ch}$ with $\mathbf{A}_{td}$, $\mathbf{D}_{td_{v}}$, $\mathbf{D}_{td_{e}}$ and $\mathbf{E}_{td}$, respectively, in (9), we can construct a similar hypergraph convolutional network to learn disease representations through the corresponding channel. Hence, performing hypergraph convolution with both hyperedge aggregation and node aggregation steps, the HGHDA model is able to effectively capture and propagate information through the hypergraph structure. This enables HGHDA to learn meaningful and informative embeddings of herbs and diseases, considering the complex MTMC relationships between them. These embeddings are further utilized for the prediction of HDAs.

#### Model optimization

To conduct the learning process of HGHDA, the Cross-Entropy (CE) loss, denoted as $\mathscr{L}_{CE}$, is employed for training HGHDA, and it is a popular loss function indicating the error between prediction results and ground truth. The definition of $\mathscr{L}_{CE}$ is given as 


(10) \begin{align*}& \mathscr{L}_{CE}=\sum_{i,j}-[\mathbf{A}_{hd}^{ij}\cdot\ln(\boldsymbol{p}_{ij})+(1-\mathbf{A}_{hd}^{ij})\cdot\ln(1-\boldsymbol{p}_{ij})]+\lambda\|\boldsymbol{\Theta}\|_{2}^{2},\end{align*}

where $\hat{\boldsymbol{p}}_{ij}=sigmoid(\mathbf{E}_{H}^{i}{\mathbf{E}_{D}^{j}}^{T})$ is the predicted score, $\mathbf{E}_{H}=\frac{1}{l}\sum _{i=1}^{l}\mathbf{M}_{ch}^{i}$, $\mathbf{E}_{D}=\frac{1}{l}\sum _{i=1}^{l}\mathbf{M}_{td}^{i}$, $l$ is the number of layers of the convolutional network and $\boldsymbol{\Theta }$ is the trainable parameter set of HGHDA, $\mathbf{A}_{hd}$ is the association matrix of herbs and diseases. In the construction of $A_{hd}$, we specify that the value of each element is either 0 or 1. Specifically, if a herb is associated with a disease, the corresponding element in $A_{hd}$ is set to 1, and 0 otherwise. It is important to note that there is no bias in $A_{hd}$, as weights are not assigned to the HDAs. Each time a pair of herb and disease embeddings is fed to HGHDA, which is then optimized toward accurately identifying HDAs. In addition, the introduction of $\lambda \|\boldsymbol{\Theta }\|_{2}^{2}$ computes the $L_{2}$ regularization of $\boldsymbol{\Theta }$ to reduce generalized errors, and $\lambda $ is the hyper-parameter to adjust the impact of $\boldsymbol{\Theta }$ on the model optimization. To achieve end-to-end HDA prediction, we ultimately used an inner-product-based scoring function $\mathscr{F}=sigmoid(\mathbf{E}_{H}\mathbf{E}_{D}^{T})$ to generate association scores between herbs and diseases.

## EXPERIMENTS

### Evaluation metrics

In order to evaluate the performance of prediction models, we begin by randomly dividing all HDAs into five equally sized folds. For each fold, an equivalent number of Herb-Disease pairs with unknown associations is randomly selected from the original $A_{hd}$ and added to that fold. In doing so, each fold is a balanced dataset, avoiding the imbalance issue. Subsequently, a cross-validation procedure is conducted by iteratively designating each fold as the testing data and the remaining folds as the training data. In our experiments, the cross-validation procedure is repeated five times for each algorithm, and the best performance is reported for comparative analysis. Several independent evaluation metrics are adopted to indicate the performance of prediction models across all folds, and they are the area under the ROC curve (AUROC), the area under the Precision-Recall curve (AUPRC) and F1-score. Among them, AUROC and AUPRC are eligible to provide valuable insight into the performance of binary classification models, and the main difference between them is that AUROC assesses the overall prediction performance across different thresholds, while AUPRC is particularly informative for imbalanced datasets. Their values range from 0 to 1, with higher ones indicating better performance. F1-socre is the harmonic mean of Precision and Recall, and it can be computed as 


(11) \begin{align*} & Recall=\frac{TP}{FN+TP} \end{align*}

(12) \begin{align*} & Precision=\frac{TP}{FP+TP} \end{align*}

(13) \begin{align*} & F1-score=\frac{2 \ast Precison \ast Recall}{Precision+Recall},\end{align*}

where $TP$ is correctly predicted positive samples, $FN$ is incorrectly predicted positive samples and $FP$ is incorrectly predicted negative samples. The value of F1-score ranges from 0 to 1, where a higher value indicates better accuracy. If the F1-score is 1, it represents perfect precision and recall, while an F1-score of 0 indicates the poorest performance.

### Baseline algorithms

To validate the effectiveness of the HGHDA model, we compare its performance with several state-of-the-art models that are widely recognized in the field of herb–target protein association prediction and drug–disease association prediction. These models are chosen to serve as baselines, allowing for a comprehensive evaluation regarding the performance of HGHDA.

HTInet [35] is a prediction model that operates on heterogeneous networks. It constructs a network by considering the correlations between various biological molecules, and employs the node2vec algorithm to learn feature representations of both herbs and target proteins within this network. The features of herb–target protein pairs are derived using the Hadamard product, a mathematical operation that involves element-wise multiplication of corresponding elements in two matrices. These features are then used to train a classifier, which in turn predicts potential HDAs based on their features. In the context of experimentation, we have developed two variations of HTInet: HTInet-KNN and HTInet-RF, which employ k-nearest neighbors (KNN) and random forest (RF) classifiers, respectively, for predicting HDAs.

BiGI [51] employs bipartite graphs, global and local features and a mutual information maximization approach to learn and represent associations between entities. Using a logistic regression classifier, the model makes predictions based on the association features. This combination allows BiGI to effectively capture relationships and patterns within complex bipartite graphs for accurate association prediction.

SMGCL [52] is a specialized model designed for drug–disease association prediction. It operates on the basis of similarity measures, and employs a unique approach called graph co-contrastive learning to process the information from distinct local and global viewpoints. This allows the model to capture intricate relationships between drugs, diseases and their associations for prediction.

MilGNet [53] is an advanced approach for predicting drug–disease associations, specifically focusing on heterogeneous graphs. It leverages heterogeneous graph structures, metapath-based learning and attention mechanisms for the prediction of drug–disease associations. By considering multiple scales and relationship types, the model aims to provide accurate and interpretable predictions, making it a useful tool for identifying novel drugs of disease treatment.

LHGCE [54] is an end-to-end model that establishes multiple networks based on the correlations between different biological entities and combines them into a single heterogeneous network. The features of nodes in the network are learned using a multilayer heterogeneous graph convolutional encoder, and the embeddings corresponding to HDAs are fed into a linear layer to generate labels for the associations.

HGNNLDA [55] is a model for sensitivity prediction between long noncoding RNA (lncRNAs) and drugs using hypergraph neural networks, which first obtains the higher order neighboring information of lncRNAs and drugs through hypergraph neural networks, then generates embeddings of lncRNAs and drugs using the joint updating mechanism, and finally obtains the sensitivity level between lncRNAs and drugs using the inner product.

### The performance of HGHDA

The effectiveness of HGHDA is evaluated by conducting a comprehensive evaluation using the TCM-suite dataset. By combining herb–component, component–target protein and target protein–disease associations, we construct a hypergraph for predicting potential HDAs. The evaluation has been performed using a 5-fold CV approach, which ensures a robust assessment for the performance of HGHDA.

The specific results of CV experiments are presented in Table 2, which provides detailed insights into the performance of HGHDA across all folds and various evaluation metrics. From the results, it is evident that the HGHDA model has achieved outstanding performance across multiple metrics, including F1-score, AUROC and AUPRC.

**Table 2:** Five-fold cross-validation results of HGHDA on the TCM-suite dataset

Fold	Recall	Precision	F1-score	AUROC	AUPRC
1	0.9681	0.9676	0.9678	0.9924	0.9898
2	0.9697	0.9641	0.9669	0.9920	0.9896
3	0.9693	0.9671	0.9682	0.9920	0.9893
4	0.9695	0.9653	0.9674	0.9921	0.9896
5	0.9672	0.9675	0.9673	0.9921	0.9897
Avg	0.9688	0.9663	0.9675	0.9921	0.9896

The average performance of HGHDA across each metric is impressive, consistently exceeding $96\%$, with notable performance in AUROC where the average is above $99\%$. This indicates that HGHDA excels in accurately predicting both positive and negative samples, as well as maintaining a high level of discrimination between positive and negative samples. Moreover, the minor differences observed in each metric across different folds suggest that the performance of HGHDA is stable and consistent.

The high recall and precision values further demonstrate the capability to effectively differentiate between relevant and irrelevant associations, resulting in a low rate of misclassifications. This collectively showcases the excellent predictive ability of the HGHDA model.

In summary, the experimental results are a strong indicator that the HGHDA model has demonstrated remarkable performance in predicting potential HDAs. The strong outcomes across various evaluation metrics, along with the stability of the performance, underscore its effectiveness on the task of HDA prediction.

### Comparison with state-of-the-art models

A comprehensive comparison between the HGHDA model and several advanced methods is given in this section in terms of their ability to predict HDAs. These baseline models include HTInet, BiGI, SMGCL, MilGNet, LHGCE and HGNNLDA. We re-implement them by removing their own cross-validation procedures. Consequently, these algorithms can now adhere to the same cross-validation procedure as HGHDA, ensuring identical data splits. For all algorithms, each is further validated by repeating the cross-validation procedure five times. This approach allows us to evaluate how well HGHDA performs in comparison with these well-established algorithms.

**Table 3:** Performance evaluation of all algorithms on two benchmark datasets

Dataset	Metric	Method							
		HTInet-KNN	HTInet-RF	BiGI	SMGCL	HGNNLDA	MilGNet	LHGCE	HGHDA
TCM-suite	Recall	0.9983($\pm $0.0003)	0.9340($\pm $0.0027)	0.8751($\pm $0.0053)	0.8644($\pm $0.0054)	0.8571($\pm $0.0035)	0.9264($\pm $0.0044)	0.9559($\pm $0.0020)	0.9688($\pm $0.0007)
	Precision	0.5010($\pm $0.0002)	0.5276($\pm $0.0009)	0.6549($\pm $0.0147)	0.7029($\pm $0.0102)	0.7362($\pm $0.0051)	0.8888($\pm $0.0038)	0.9068($\pm $0.0019)	0.9663($\pm $0.0010)
	F1-score	0.6667($\pm $0.0001)	0.6743($\pm $0.0002)	0.7490($\pm $0.0108)	0.7752($\pm $0.0049)	0.7920($\pm $0.0019)	0.9072($\pm $0.0039)	0.9307($\pm $0.0019)	0.9675($\pm $0.0003)
	AUROC	0.5684($\pm $0.0003)	0.6483($\pm $0.0008)	0.8010($\pm $0.0155)	0.8339($\pm $0.0048)	0.8532($\pm $0.0024)	0.9712($\pm $0.0022)	0.9780($\pm $0.0010)	0.9921($\pm $0.0001)
	AUPRC	0.5609($\pm $0.0001)	0.6393($\pm $0.0009)	0.7922($\pm $0.0161)	0.8163($\pm $0.0048)	0.8367($\pm $0.0029)	0.9676($\pm $0.0022)	0.9751($\pm $0.0012)	0.9896($\pm $0.0006)
Ethnobotany	Recall	0.9956($\pm $0.0017)	0.8918($\pm $0.0140)	0.7955($\pm $0.0188)	0.9921($\pm $0.0025)	0.7619($\pm $0.0104)	0.8248($\pm $0.0127)	0.8191($\pm $0.0071)	0.8618($\pm $0.0087)
	Precision	0.5016($\pm $0.0006)	0.5566($\pm $0.0057)	0.7318($\pm $0.0158)	0.5048($\pm $0.0010)	0.7913($\pm $0.0133)	0.7980($\pm $0.0180)	0.7763($\pm $0.0080)	0.8196($\pm $0.0079)
	F1-score	0.6671($\pm $0.0003)	0.6852($\pm $0.0018)	0.7616($\pm $0.0019)	0.6691($\pm $0.0006)	0.7760($\pm $0.0024)	0.8109($\pm $0.0110)	0.7970($\pm $0.0021)	0.8400($\pm $0.0020)
	AUROC	0.5707($\pm $0.0026)	0.7218($\pm $0.0023)	0.8301($\pm $0.0054)	0.5367($\pm $0.0069)	0.8454($\pm $0.0016)	0.8793($\pm $0.0136)	0.8329($\pm $0.0021)	0.9028($\pm $0.0006)
	AUPRC	0.5785($\pm $0.0025)	0.7529($\pm $0.0023)	0.8423($\pm $0.0082)	0.5260($\pm $0.0051)	0.8709($\pm $0.0024)	0.8886($\pm $0.0175)	0.8265($\pm $0.0024)	0.9033($\pm $0.0013)

Note:Best results are bolded.

**Figure 2:**
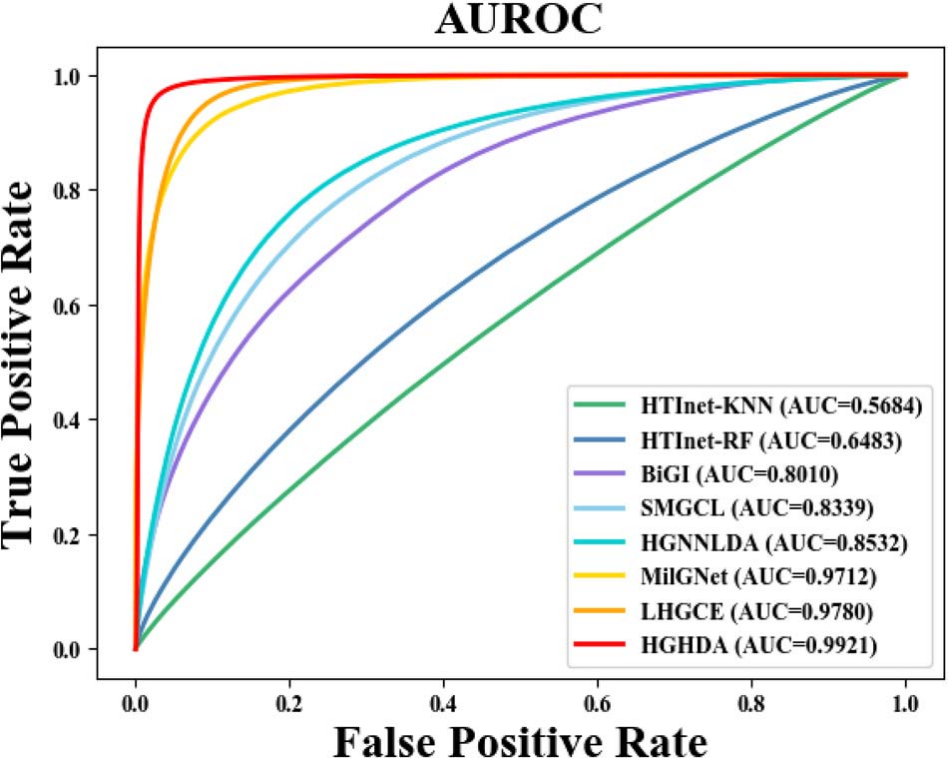
AUROC of different methods on the TCM-suite dataset.

**Figure 3:**
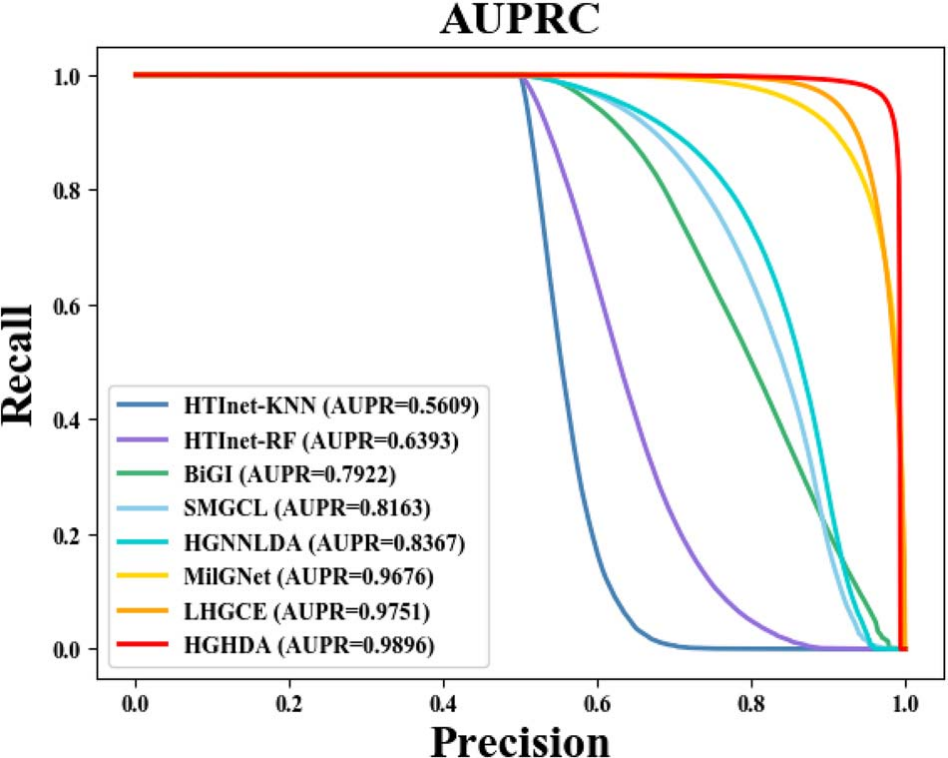
AUPRC of different methods on the TCM-suite dataset.

The specific results of these comparison experiments are presented in Table 3, Figure 2 and Figure 3. When evaluating the performance of all algorithms on the TCM-suite dataset, we note that the performance of HGHDA surpasses that of all the compared algorithms. Notably, HGHDA outperforms HTInet by more than 30$\%$. The better performance of HGHDA might be attributed to its unique design, which leverages hypergraph convolution to effectively capture high-order information so as to preserve essential features of herbs and diseases. For HTInet, using the RF classifier for binary classification performs better than using KNN. However, HGHDA outperforms HTInet significantly. The discrepancy in performance might stem from the way HTInet constructs associative embeddings using Hadamard products and network embedding representations. Compared with BiGI using bipartite graph representation, HGHDA achieves about 19$\%$ improvement in AUROC and AUPRC metrics and over 21$\%$ improvement in F1-score. This suggests that hypergraphs, which HGHDA utilizes, are better suited for capturing multi-to-multi relationships compared with ordinary graphs. The performance of SMGCL, based on similarity graph Co-contrastive learning, is better than HTInet and BiGI, indicating the effectiveness of graph co-contrastive learning for predicting HDAs. However, it lags slightly behind graph convolutional network models like MilGNet and HGHDA. This might be due to the superior capability of graph convolutional models to analyze graph topology. As a graph-convolution-based model, MilGNet achieves the third-best performance among all models. While the AUROC score of HGHDA is only around 2$\%$ higher than MilGNet, HGHDA significantly improves Recall, Precision and F1-score by about 12$\%$ on average than MilGNet. This again indicates the efficacy of hypergraphs in capturing higher order structures compared with ordinary graphs. In a comprehensive evaluation across various metrics, HGHDA consistently outperforms both HGNNLDA and LHGCE, indicating its strong potential as a valuable tool for identifying novel HDAs. Despite the innovative use of hypergraph neural networks to leverage higher order neighboring information, the performance of HGNNLDA lags behind that of HGHDA by a noticeable 14$\%$. This discrepancy may be attributed to the hypergraph structure employed by HGNNLDA, which might not address the intricate MTMC mechanisms in HDA discovery. Regarding two graph convolution network models, i.e. LHGCE and MilGet, their performance surpasses that of other baseline algorithms but falls short of HGHDA. Both LHGCE and MilGet construct heterogeneous networks by integrating diverse associations, and employ a single neural network to learn node embeddings. In contrast, HGHDA adopts a unique approach, obtaining embeddings for herbs and diseases through two separate channels, enabling the capture of their distinct characteristics.

**Table 4:** Experimental results of ablation study on the TCM-suite dataset

Method	Recall	Precision	F1-score	AUROC	AUPRC
HGHDA-m	0.8235	0.7284	0.7730	0.8324	0.8400
	($\pm $0.0059)	($\pm $0.0039)	($\pm $0.0024)	($\pm $0.0024)	($\pm $0.0026)
HGHDA-s	0.9587	0.9528	0.9558	0.9884	0.9802
	($\pm $0.0012)	($\pm $0.0008)	($\pm $0.0003)	($\pm $0.0001)	($\pm $0.0007)
HGHDA-t	0.9312	0.9224	0.9268	0.9776	0.9758
	($\pm $0.0022)	($\pm $0.0035)	($\pm $0.0027)	($\pm $0.0015)	($\pm $0.0017)
HGHDA-b	0.8870	0.8081	0.8457	0.9215	0.9180
	($\pm $0.0035)	($\pm $0.0087)	($\pm $0.0056)	($\pm $0.0052)	($\pm $0.0055)
HGHDA-c	0.9537	0.8944	0.9230	0.9376	0.9719
	($\pm $0.0088)	($\pm $0.0090)	($\pm $0.0044)	($\pm $0.0032)	($\pm $0.0017)
HGHDA	0.9688	0.9663	0.9675	0.9921	0.9896
	($\pm $0.0007)	($\pm $0.0010)	($\pm $0.0003)	($\pm $0.0001)	($\pm $0.0006)

Note: best results are bolded.

We then proceed to perform a cross-validation experiment on the Ethnobotany dataset, presenting the results in Table 3. It is noteworthy that HGHDA consistently outperforms all compared algorithms in terms of F1-score, AUROC and AUPRC. Across all metrics, HGHDA exhibits improvements of 31%, 20%, 9%, 34%, 7%, 3% and 7% compared with HTInet-KNN, HTInet-RF, BiGI, SMGCL, HGNNLDA, MilGNet and LHGCE, respectively. These results strongly affirm HGHDA’s proficiency in uncovering new HDAs.

It is worth noting that due to the complex mechanisms involved in herb treatments for diseases, there are very few computational models specifically designed for predicting novel HDAs. Among the baseline algorithms, SMGCL and MilGNet are primarily developed for drug repurposing, whereas the rest are general-purpose graph representation learning models. In this regard, the exceptional performance of HGHDA is primarily attributed to its unique integration of MTMC mechanisms through a dual-channel hypergraph convolution network. This alignment with the nature of herb treatments for diseases significantly improves the accuracy of HGHDA in predicting HDAs. In conclusion, HGHDA stands out as a powerful approach for HDA prediction. Its ability to leverage hypergraph convolution effectively captures high-order information and important features, leading to improved performance compared with other advanced models. Our experimental results underscore the ability of HGHDA to predict HDAs by effectively utilizing hypergraphs and achieving accurate results.

### Ablation study

To thoroughly investigate the impact of hyperedge embedding aggregation and node initialization on model performance, the study introduced four variants of the HGHDA model: HGHDA-m, HGHDA-s, HGHDA-t and HGHDA-b. Their detailed descriptions are given as below.

- HGHDA-m: in this variant, HGHDA employs the average pooling to obtain the final embeddings of herbs and diseases from different layers of the convolutional network, rather than summing up the embeddings, as done in the original HGHDA model.
- HGHDA-s: for this variant, the model diverges from the original initialization process. More specifically, HGHDA-s randomly initializes the embeddings of components and target proteins.
- HGHDA-t: for this variant, we use the tanimoto coefficient to calculate the similarity between the components, by which the initial representation of the components is obtained.
- HGHDA-b: for this variant, we use the sequence comparison matrix BLOUSUM to calculate the similarity between target protein sequences.

We then proceed to compare the performance of these variants with the original HGHDA model on the TCM-suite dataset. The results of these experiments are presented in Table 4. Here are the key observations from this comparison.

First, HGHDA-m achieves poorer results across all metrics compared with the original HGHDA model. Particularly notable is its performance in terms of Precision, as a significant difference of about 24$\%$ is observed compared with HGHDA. This finding suggests that averaging embeddings from different layers of the convolutional network might lead to the loss of unique features and information, thereby impacting the prediction performance negatively. On the other hand, the performance of HGHDA-s is improved across all metrics compared with HGHDA-m. This indicates that how to concatenate the embeddings from different layers plays a more critical role in determining the performance of HGHDA. For HGHDA-t, it can be seen that its performance is lower than HGHDA. We have chosen to utilize Dice coefficients primarily due to the high dimensionality of the generated fingerprints, in which only a few positions are set to 1. Dice coefficients are particularly suited for such high-dimensional, sparse data, as they are more sensitive to positions with values of 1. This sensitivity allows Dice coefficients to calculate molecular similarities with reduced interference, leading to more accurate results in the context of sparse, high-dimensional data. Specifically, the use of the Dice coefficient leads to a 1.4$\%$ improvement in AUROC and a 1.3$\%$ improvement in AUPRC when compared with the use of the Tanimoto coefficient. This strongly suggests that the Dice coefficient is the preferred choice for computing the similarity between herb components. For HGHDA-b, notably, the utilization of pairwise similarity, as opposed to the Blosum similarity, yields significant improvements in both AUROC and AUPRC, showing increases of 7% and 7.1%, respectively. This marked improvement can be attributed to the inherent capacity of pairwise similarity to consider the relative positions and interactions between different amino acids within a target protein sequence. The superior performance of pairwise similarity underscores its preference as the method of choice for computing the similarity of target protein. These findings emphasize the value of employing pairwise similarity, providing more accurate and informative insights for the discovery of novel HDAs. Besides, utilizing component and target protein embedding representations for initializing nodes in the hypergraph can provide valuable information for manipulating the hypergraph convolution process. This improved initialization can lead to enhanced performance by leveraging meaningful initializations rather than relying on random values.

In summary, the findings of ablation study reveal that the approach of averaging embeddings (as in HGHDA-m) could lead to a loss of information and subsequently poorer performance. However, utilizing effective embeddings for node initialization (as in HGHDA-s) could contribute to improved performance by providing valuable guidance to the hypergraph convolution process. These insights highlight the importance of careful hyperedge embedding aggregation and node initialization strategies in hypergraph-based models like HGHDA.

#### Randomly shuffled graph

**Figure 4:**
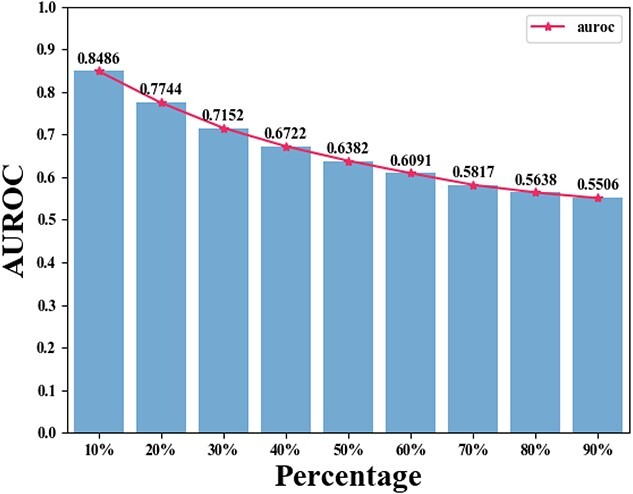
The performance of HGHDA in terms of AUROC at different randomness levels on the TCM-suite dataset.

**Figure 5:**
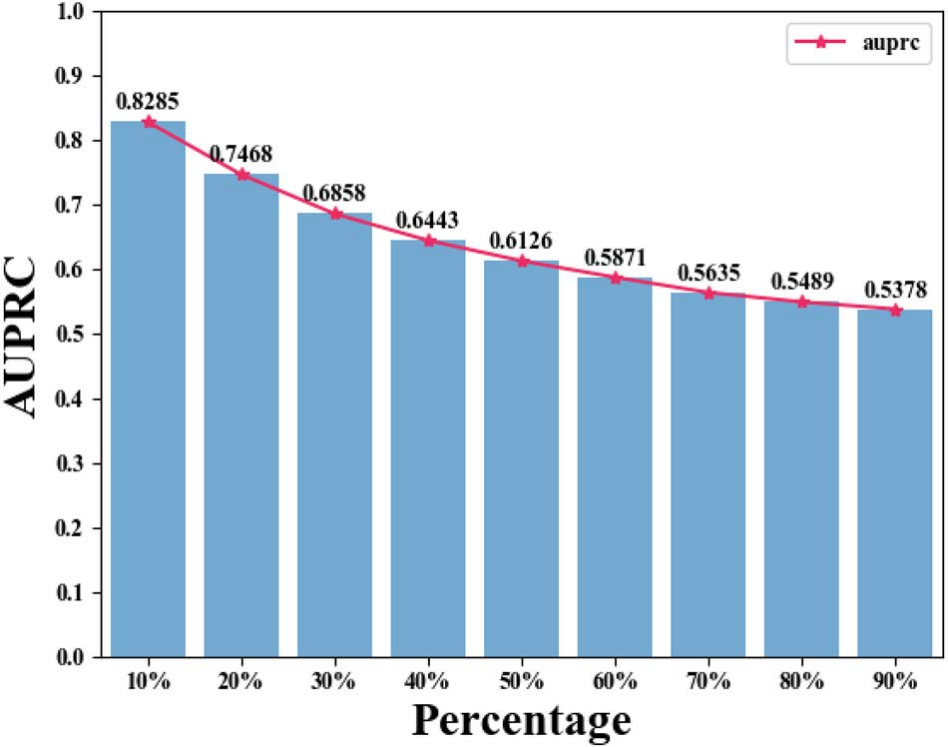
The performance of HGHDA in terms of AUPRC at different randomness levels on the TCM-suite dataset.

In our random shuffling procedure, we randomly alter a specific percentage of elements in the adjacency matrix, introducing increased randomness to the graph. This procedure is applied to $A_{ch}$, $A_{td}$ and $A_{hd}$, with the percentage value varying from the set $\lbrace 10\%, 20\%, 30\%, 40\%, 50\%, 60\%, 70\%, 80\%, 90\%\rbrace $ to construct hypergraphs at different randomness levels. For each randomness level, we conduct a 5-fold cross-validation on the randomly shuffled variants of $A_{ch}$, $A_{td}$ and $A_{hd}$, reporting the performance of HGHDA in terms of AUROC and AUPRC in Figure 4 and Figure 5, respectively. It is important to note that the performance of HGHDA decreases as the randomness level increases. Specifically, AUROC and AUPRC scores approach 0.5 when 90% of the elements in $A_{ch}$, $A_{td}$ and $A_{hd}$ are changed. This suggests that HGHDA demonstrates a robust ability to avoid overfitting to inherent dependencies in the training data, preventing it from learning specific patterns that may not generalize well. Thus, we believe that the promising performance of HGHDA in identifying novel HDAs is not a result of overfitting.

#### Cold-start case

In addition, to simulate a cold-start scenario, we randomly select a herb, and treat its HDAs as the test data. Subsequently, we train the HGHDA model using the remaining HDAs that do not involve the chosen herb. The performance of the HGHDA model is assessed by evaluating its predictive accuracy on the test data. The experimental results, presented in Table 4, clearly demonstrate the effectiveness of HGHDA-c, as evidenced by its notable accuracy across all metrics. Specifically, our analysis reveals that HGHDA-c successfully identifies 88$\%$ HDAs in the test data. These results underscore HGHDA’s capability to handle cold-start scenarios, making it a valuable tool for predicting HDAs for novel herbs or diseases.

### The sensitivity analysis of network layers

**Figure 6:**
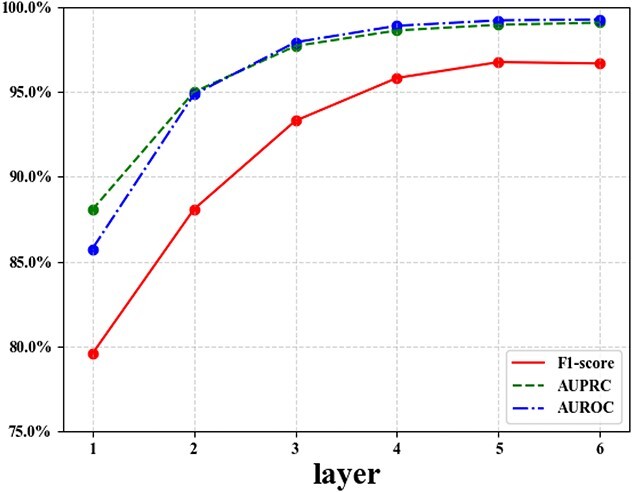
Influence of network layers on the TCM-suite dataset.

Regarding network layers, the study of sensitivity analysis delves into the impact of varying its value on the performance of HGHDA. Hence, a series of experiments have been conducted by altering the value of $l$ from the set $\left \{1, 2, 3, 4, 5, 6\right \}$. The results corresponding to different values of $l$ are evaluated based on AUROC, AUPRC, F1-score and computation time. The summarized results are presented in Figure 6.

**Figure 7:**
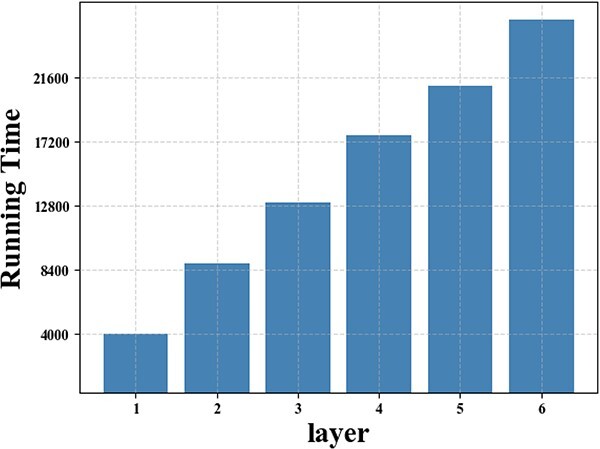
Running Time of HGHDA given different numbers of network layers on the TCM-suite dataset.

As the number of network layers increases, the performance of HGHDA gradually improves. Such improvement is especially prominent when the number of network layers is fewer than three, with an average enhancement of about 7$\%$ each time. In this regard, incorporating additional layers allows the model to capture more intricate relationships and patterns, leading to improved performance. However, as the number of network layers exceeds three, the performance gain of HGHDA slows down. Beyond this threshold, the performance improvement of HGHDA becomes more trivial. When the number of network layers exceeds five, a slight reduction is observed in F1-score. This phenomenon could indicate that as the model aggregates higher order information within the hypergraph, it encounters challenges related to over-smoothing, which can lead to a decline in performance.

**Table 5:** Top 10 diseases related to CX

Rank	Disease name	Evidence	Score
1	Sleep disorders	PMID: 35769674	0.971
2	plasma levels of liver enzymes	PMID: 23895155	0.957
3	infantile hypophosphatasia	-	0.956
4	hemoglobin	PMID: 20309798	0.955
5	liver enzymes	PMID: 28056664	0.955
6	Rhinitis	PMID: 35850854	0.954
7	radiotherapy response	PMID: 33967762	0.954
8	chondrocalcinosis	-	0.954
9	folate pathway vitamin levels	-	0.954
10	oral cancer	PMID: 15339418	0.952

Note:- indicates that no relevant literature was found.

In addition to the accuracy of HGHDA, we also study the impact of network layers from the computational aspect. It is evident from Figure 7 that the computation time increases with the growing number of network layers. This trend is expected, as more layers lead to more computations. Hence, given the balance between performance enhancement and computation time, we conclude that the optimal number of network layers for the model is recommended as five. This configuration yields a satisfactory level of performance improvement while maintaining manageable computation time.

### Case study

In the case study, we employ the HGHDA model to reposition two traditional herbs: Chuan Xiong (CX) and Astragalus membranaceus (AM). To train the HGHDA model, we first remove all herb-disease pairs that involve either CX or AM from the TCM-suite dataset, and then regard the rest as the training data. Once trained, HGHDA is then used to predict unknown diseases associated with CX and AM. Ranked in the descending order of prediction scores, top 10 diseases are listed in Table 5 and Table 6 for each herb. Remarkably, seven and eight out of the top 10 diseases associated with CX and AM, respectively, are validated by relevant literature. This high validation rate demonstrates the promising accuracy of HGHDA in predicting potential associations between herbs and diseases. A detailed discussion is provided as follows.

**Table 6:** Top 10 diseases related to AM

Rank	Disease name	Evidence	Score
1	sleep	PMID: 36801290	0.967
2	Lithium response	-	0.942
3	Stroke	PMID: 34044075	0.941
4	sickle cell	-	0.941
5	nicotine dependence	PMID: 15844840	0.938
6	Hypertension	PMID: 36119929	0.936
7	Acute lymphoblastic leukemia	PMID: 24568907	0.935
8	Respiratory Distress Syndrome	PMID: 35401158	0.933
9	eosinophilia	PMID: 25264079	0.932
10	Apolipoproteins	PMID: 36148321	0.932

Note:- indicates that no relevant literature was found.

Chuanxiong is commonly used to treat cardiovascular, cerebrovascular diseases and headaches. Chuanxiong contains components such as chuanxiongzine and chuanxiongone which are used for anti-inflammatory, antioxidant and also for nerve protection. By regulating the body’s endocrine system, chuanxiong can be used to treat sleep disorders to some extent [56]. Due to the antioxidant properties of the active ingredients in Chuanxiong, it is able to protect the liver by increasing the level of antioxidant enzymes in the liver [57, 58]. When Chuanxiong is used together with Angelica, in addition to stimulating blood circulation it also induces the production of red blood cells [59]. Chuanxiong contains ligusticum lactone, which can prevent and treat radiation-induced enteritis by improving intestinal ischemia [60]. As for Astragalus membranaceus, its bioactive components include astragalus polysaccharides (APS), which have antioxidant, neuroprotective and anti-cancer effects. APS can also reduce sleep disorders caused by aging [61]. The antioxidant activity of Astragalus membranaceus can accelerate the conversion of nicotine to alleviate the symptoms of quitting smoking and reduce dependence on nicotine [62]. Astragalus saponins in Astragalus membranaceus have neuroprotective effects and can promote the proliferation of neural stem cells by target proteining Akt target proteins to treat stroke [63]. Astragalus membranaceus combined with Salvia miltiorrhiza and Panax notoginseng can reduce serum triglycerides, cholesterol, and treat hypertension [64]. Astragalus membranaceus relieves respiratory distress by inhibiting pro-inflammatory cytokines and decreasing eosinophil levels [65, 66].

**Figure 8:**
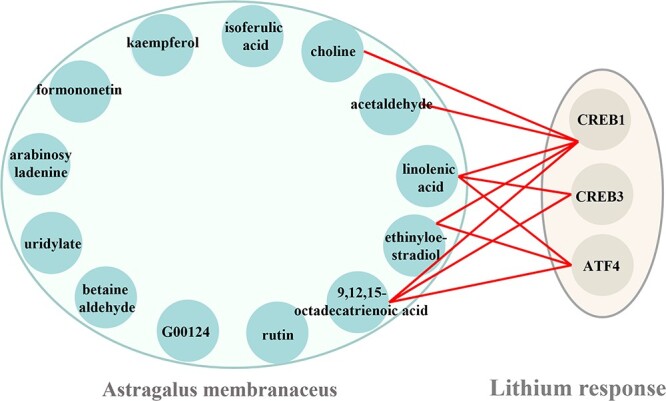
Relationship between Astragalus membranaceus and Lithium response.

In addition to herb–disease pairs that are validated in the relevant literature, there are also potential relationship between those that are not validated. From the perspective of the MTMC and Figure 8, the relationship between AM and lithium response pair is illustrated. ATF4, CREB1 and CREB3 are three target proteins related to lithium response, and the ethynylestradiol and linolenic Acid in AM may affect the levels of the response elements related to them by influencing the intracellular signaling pathway, thus affecting their activities and ultimately influencing the LR. ALPL is associated with several biometabolic pathways. Infantile hypophosphatasia, chondrocalcinosis and folate pathway vitamin levels are all related to ALPL. Various active components contained in CX, such as Thiamine monophosphate, may affect ALPL through energy metabolism and related neurological pathways, caffeic acid and 2,4-Dihydroxyacetophenone may affect ALPL activity by regulating the intracellular environment due to their antioxidant properties. This in part illustrates the superior ability of HGHDA to predict potential herb–disease pairs through the use of MTMC mechanisms.

The high accuracy achieved by the HGHDA model in repositioning CX and AM highlights its effectiveness in predicting novel HDAs. The fact that the prediction results of HGHDA are consistent well with existing literature further reinforces its credibility and utility. According to our case study, the HGHDA model demonstrates its ability to effectively reposition herbs for potential disease treatments. The validation of the predicted associations through relevant literature indicates the model’s reliability and practical applicability. In this regard, HGHDA can serve as a valuable tool for predicting potential HDAs and guiding traditional medicine research.

## CONCLUSION

In order to achieve HDA prediction from the perspective of molecular association and to fully utilize the multi-component and multi-target protein properties of herbs, we propose a dual-channel hypergraph convolutional network model named HGHDA to achieve HDA prediction. HGHDA converts the herb–component association and target protein–disease association into different hypergraphs and sends them to different channels for hypergraph convolution. The convolution process aim to obtain herb and disease embeddings and predict potential HDA. Compared with some other existing methods, HGHDA uses molecular association information between herb and disease, and HGHDA uses hypergraph to better obtain multi-component and multi-target association information. In addition, experimental results show that HGHDA outperforms some existing state-of-the-art algorithms. Although the experimental results show that HGHDA can be used well for HDA prediction tasks, there is still some room for improvement, and we consider using more biological knowledge (MeSH of diseases), introducing more biological association information (e.g. prescription–herb association, herb–herb association, etc.) to enrich the features of herbs and diseases. Or in the future use feature fusion to fuse multiple features of herbs and diseases to better enable herb repositioning.

**Key Points**

- Most of the existing association prediction methods related to herbs predict the relationships between component in herbs and target protein or the relationship between herbs and target proteins; there are few methods that directly perform HDA prediction. In this paper we propose a dual-channel hypergraph convolution-based model for HDA prediction.
- To better obtain the multi-component and multi-target association information of herbs, this paper first uses the embedding of component and target protein to initialize the component and target protein nodes in the hypergraph; then it sends different hypergraphs to different channels, and uses skip connections in the hypergraph convolution to obtain the embeddings of herbs and diseases.
- Based on the embeddings of herbs and diseases learned from hypergraphs, HGHDA achieves good performance on two benchmark datasets and outperforms state-of-the-art prediction algorithms in terms of F1-score, AUROC and AUPRC. In addition, the repositioning results for Chuan Xiong and Astragalus membranaceus shows that HGHDA can perform the HDA prediction task well.

## Data Availability

The dataset and source code can be freely downloaded from https://github.com/bioxjz/HGHDA.

## Notes

**Lun Hu** is a Professor of Xinjiang Technical Institute of Physics & Chemistry, CAS. His research interests include machine learning, complex network analytics and their applications in Bioinformatics.

**Menglong Zhang** is a Master Degree candidate at Xinjiang Technical Institute of Physics & Chemistry, CAS. His research interests include bioinformatics, machine learning and deep learning.

**Pengwei Hu** is a Professor of Xinjiang Technical Institute of Physics & Chemistry, CAS. His research interests include data mining, machine learning, computational biology and bioinformatics.

**Jun Zhang** is an engineer of computer science at Xinjiang Technical Institute of Physics & Chemistry, CAS. His research interests include data mining and natural language processing.

**Chao Niu** is an Associate Professor of Xinjiang Technical Institute of Physics & Chemistry, CAS. His research interests include natural product discovery and computer-aided drug design.

**Xueying Lu** is an Associate Professor of Xinjiang Technical Institute of Physics & Chemistry, CAS. Her research interests include pharmacology and therapeutic efficacy evaluation of drugs.

**Xiangrui Jiang** is a Professor of Shanghai Institute of Materia Medica, CAS. His research interests include the discovery of antiviral drugs.

**Yupeng Ma** is a Professor of Xinjiang Technical Institute of Physics & Chemistry, CAS. His research interests include internet of things and big data analysis.
